# Secondary Traumatic Stress and Vicarious Posttraumatic Growth among nurses during three COVID-19 lockdowns in Greece

**DOI:** 10.1192/j.eurpsy.2022.795

**Published:** 2022-09-01

**Authors:** A. Kalaitzaki, G. Tsouvelas, A. Tamiolaki, M. Theodoratou, G. Konstantakopoulos

**Affiliations:** 1 Hellenic Mediterranean University, Department Of Social Work, Heraklion, Greece; 2 University of West Attica, Department Of Nursing, Athens, Greece; 3 Hellenic Open University, School Of Social Sciences, Patra, Greece; 4 National and Kapodistrian University of Athens, Department Of Psychiatry, Athens, Greece

**Keywords:** coping strategies, Vicarious Posttraumatic Growth, Covid-19 pandemic, Secondary Traumatic Stress

## Abstract

**Introduction:**

Since the onset of the pandemic, nurses have been repeatedly exposed to their patients’ COVID-19-related traumatic experiences. Therefore, they are at high risk for Secondary Traumatic Stress (STS), the stress syndrome resulting from helping others who are suffering. Positive psychological outcomes following this vicarious exposure are also likely. Vicarious posttraumatic growth (VPTG) refers to the positive changes from working with patients who themselves have coped with traumatic experiences.

**Objectives:**

This study aims to examine STS and VPTG among 429 nurses during three lockdowns of the COVID-19 pandemic in Greece.

**Methods:**

A repeated cross-sectional survey with a convenience and snowball sampling procedure was conducted. The Secondary Traumatic Stress Scale (STSS), the Post-Traumatic Growth Inventory (PTGI), the Brief Resilience Scale, and the Brief Cope (BC) were used to measure STS, VPTG, resilience, and coping strategies, respectively.

**Results:**

Nurses in Greece demonstrated high levels of STS at the first lockdown, significantly lower in the second one, which raised again -but not significantly- in the third lockdown. Resilience significantly decreased, whereas VPTG significantly increased across the three lockdowns. Following the escalation of the pandemic nurses in general used significantly more adaptive and less maladaptive coping strategies to deal with the crisis.

**Conclusions:**

Further research is needed to clarify the longitudinal course of the negative and positive psychological effects of the pandemic on healthcare staff. Conclusions can guide the development of interventions to safeguard nurses from the deleterious impacts of the COVID-19 and support them in their process of growth.

**Disclosure:**

No significant relationships.

